# From line to dots: an improved computerised rod and frame system for testing subjective visual vertical and horizontal

**DOI:** 10.1186/1756-0500-3-9

**Published:** 2010-01-19

**Authors:** Sharon Docherty, Jeff Bagust

**Affiliations:** 1 AECC, 13-15 Parkwood Road, Bournemouth, BH5 2DF, UK

## Abstract

**Background:**

Perception of subjective visual vertical (SVV) and horizontal (SVH) has traditionally been measured by rotating a mechanical rod either with or without a frame present. The computerised rod and frame (CRAF) system has previously only been used to measure SVV. We have expanded the use of this system by testing its feasibility to measure SVH. This was done by comparing two groups of subjects (n = 103) randomly assigned to be tested for SVV or SVH.

**Findings:**

Preliminary results showed a higher than expected percentage of individuals with SVH errors < 0.5°. This was attributed to additional visual cues provided by the changing appearance of the rod as it approached the horizontal. A solution to this problem was sought by replacing the rod by two dots to mark its ends. In a second investigation 30 subjects were tested using both the "rod as line" and "rod as dots" presentation. Bland and Altman analysis showed no difference between the rod and dots presentations in the measurement of SVV, but confirmed a fixed error of -0.93° between rods and dots for SVH. Changing the rod from a line to dots in the computer system resulted in errors for both SVV and SVH that were comparable to previous studies using manual systems.

**Conclusions:**

The computerized rod and frame system may be improved by replacement of the line with two dots. This reduces clues provided to the subject by the appearance of the rod on the screen.

## Background

Measurement of subjective visual vertical (SVV) or horizontal (SVH) using the rod and frame test, pioneered by Witkin [1], is an established way of investigating spatial orientation [2-7]. The test is simple but a number of different systems have been used. In some studies a light emitting rod [2,3,8,9], or a rod and a frame [10-15] have been adjusted using a mechanical device, in others they have been projected onto a screen [16]. In the majority of cases a completely dark room and specialised laboratory facilities are required.

The Computerised Rod and Frame (CRAF) Test was developed to make measuring visual perception more flexible [17]. Previous studies have investigated the range of errors in perception of SVV using this system under traditional dark laboratory conditions [17] and also in a less controlled setting [18]. The aim of the current study was to explore the feasibility of using the CRAF to measure SVH and to establish the range of errors that can be considered normal for both SVH and SVV.

Comparison of the errors generated in horizontal and vertical tests revealed significantly smaller errors in the horizontal plane which may have resulted from changes in the appearance of the rod as it approached horizontal. To eliminate this, the rod was changed from a line to two dots and more subjects tested.

## Methods

### Participants

Healthy volunteers were recruited from the staff and students of the Anglo-European College of Chiropractic (AECC). The study was approved by the AECC Research Ethics Committee. All subjects gave written informed consent prior to taking part. Subjects had normal or corrected to normal vision. There were no time constraints and the head was not restrained during the task.

### The CRAF Test

Subjects completed the test seated in a room with subdued illumination. Participants performed the CRAF test wearing Olympus Eye-Trek FMD 200 video eyeglasses which had a viewing angle of 30° × 23° (horizontal × vertical) and gave the impression of viewing a large screen (width 142 cm), from a distance of 2 m. Where necessary the video eyeglasses were used over spectacles.

**Figure 1:**
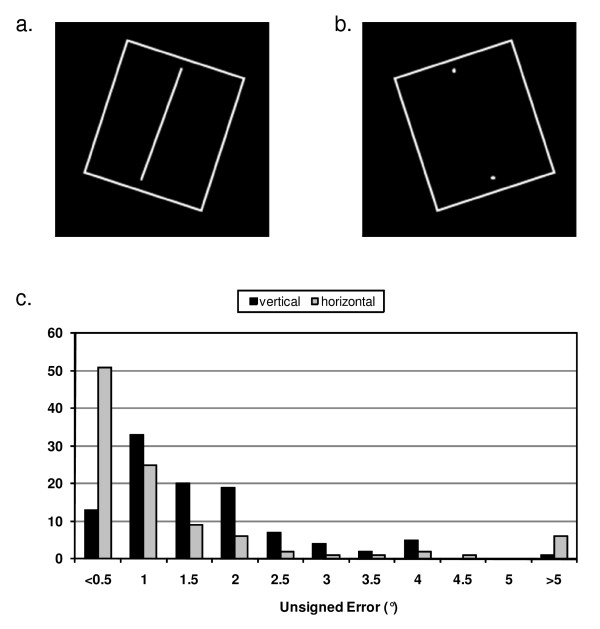
**The computer rod and frame test**. a) Screen display with rod as line, frame tilted +18°, rod +20°; b) Rod as dots display, frame tilted -18°, rod -20°; c) Distribution of unsigned positioning errors to vertical and horizontal with rod as line (n = 104).

The test was presented as a white square (the frame), positioned centrally, surrounding a white rod on a homogeneous black background (Figure 1a). The first two presentations in each set were for instruction and were not included in the analysis. For the remaining presentations, the frame orientation was either untilted (0°, frame°), tilted clockwise (+18°, frame^+18^) or tilted counterclockwise (-18°, frame^-18^). There were also two starting positions for the rod which was tilted clockwise (+20°) or counterclockwise (-20°). Each frame presentation was replicated four times in a random order assigned by the computer.

The task was to rotate the rod using the mouse buttons to a position perceived as vertical or horizontal depending on the test. The rod rotated around its centre in 0.5° increments (movement resolution: horizontal 8 pixels/deg; vertical 6 pixels/deg). When the subject was satisfied with the rod alignment, the programme was advanced by pressing the spacebar. The measured deviation (degrees) of the perceived position from gravitational vertical/horizontal was recorded by the programme.

### Rod as Line

One hundred and three volunteers were recruited for the first part of the investigation in which the rod was represented by a line. Subjects were randomly assigned to either the SVH or SVV groups. The test consisted of 14 presentations of the rod and frame of which presentations 3-14 were included in the analysis.

### Line versus Dots

Participants for this part of the study were recruited separately for the SVH (30) and SVV (30) trials. During these tests participants were shown 26 presentations of the rod and frame which consisted of two practice screens and 12 screens where the rod was a line, randomly interspersed with 12 screens where the subjects were told that the two white dots marked the ends of a line (Figure 1b).

### Statistical Analysis

Recorded errors were used to calculate the signed and unsigned (absolute) means for the three frame orientations (n = 4 in each case) for each participant. The signed mean error was used to indicate the overall direction of the deviation while the unsigned mean error gives an indication of its magnitude and spread. All statistical analyses were performed using SPSS 16.0. Data were tested for normality using the one sample Kolmogorov-Smirnov test. Unless otherwise stated, data are given as mean ± standard deviation (SD). Bland and Altman analysis [19] was used to investigate the method agreement between the two rod conditions (line or dot) for both SVV and SVH.

## Results

### Visual Vertical - Rod as Line

Fifty one participants were randomly selected for the SVV test (27 male). Ages ranged from 18 to 61 (34.7 ± 13.8 years).

The signed perceptual error for each frame orientation followed a normal distribution. With the frame° orientation the mean error was close to zero and increased when the frame was tilted, moving in the direction of the frame tilt (Table 1).

**Table 1:** Mean and median errors for vertical and horizontal tasks

		Frame^-18^		Frame°		Frame^+18^	
		Mean ± SD	Median	Mean ± SD	Median	Mean ± SD	Median
Vertical (n = 51)	S	-1.52 ± 1.61	-1.44	-0.24 ± 0.44	-0.25	0.87 ± 1.16	0.69
	US	1.26 ± 0.75	1.63	0.44 ± 0.31	0.38	1.34 ± 1.03	1.00
Horizontal (n = 52)	S	-0.99 ± 3.31	-0.25	0.14 ± 0.29	0.07	0.12 ± 4.76	0.63
	US	1.20 ± 3.25	0.50	0.32 ± 0.24	0.38	1.96 ± 4.43	0.71

Comparison of mean and median signed (S) and unsigned (US) errors (degrees from gravitational vertical and horizontal) for rod as line, SVH versus SVV (different individuals).

A similar pattern was seen for the unsigned errors. The smallest unsigned errors were found with the frame° orientation (maximum = 2°, median = 0.38°). In both the frame^-18 ^and frame^+18 ^conditions the maximum observed unsigned error increased (frame^-18 ^9.50°; frame^+18 ^3.88°) with corresponding increases in the median values (Table 1). Figure 1c shows the distribution of the unsigned errors for the combined frame^+18 ^and frame^-18 ^values.

### Visual Horizontal - Rod as Line

There were 52 participants in the SVH test (27 male), mean age 31.7 ± 11.0 years (range 18-62). None of the distributions for mean signed errors was normal with a clustering of errors between -1° and +1°. For frame° the median error was 0.07° (range: -0.38° to 1.00°). Both the frame^-18 ^and frame^+18 ^median errors (-0.25°, range: -5.13° to 1.13°; and 0.63°, range: -0.5° to 5.5° respectively) were smaller than the corresponding values in the SVV data (Table 1).

The maximum unsigned error for the frame° condition was 1° with a median of 0.38°. For frame^+18 ^the maximum was 5.50° with a median of 0.71° and the frame^-18 ^condition had a maximum of 5.13° with a median of 0.50°. Notably 49% of the unsigned errors were less that 0.5° (one mouse button press), compared to 12.5% in the vertical test (Figure 1c).

Sixteen of the 52 participants reported that the line had a stepped appearance that changed to smooth when the line was horizontal. A second investigation was therefore undertaken to determine if replacing the line with two dots marking its ends would remove this bias.

### Rod as Line versus Dots - Vertical

**Figure 2:**
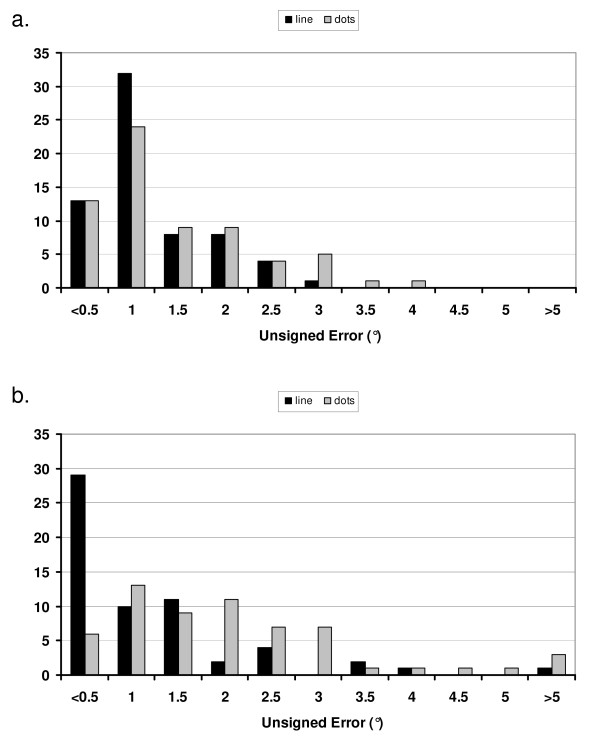
**Comparison of line and dots**. a) Distribution of unsigned errors in setting rod to vertical (n = 66); b) distribution of absolute errors in setting rod to horizontal with rod as dots, (n = 60). In both cases black bars = rod as line, grey bars = rod as dots.

Of 30 individuals aged 20-62 years (mean 33.1 ± 13.1 years) recruited to this investigation, 15 were male. All of the errors followed a normal distribution. Table 2 shows the mean (± SD) signed and unsigned errors for the lines and dots under all frame conditions. A two-sample Kolmogorov-Smirnov test showed that the distribution of these line results for frame tilted conditions did not differ significantly from those found earlier when only the line was used (rod as line, frame tilted: Z = 1.04, *P *= .23). The mean values of the combined frame^+18 ^and frame^-18 ^unsigned errors with the rod as dots (1.24° ± 0.86), was significantly greater than the corresponding values for the rod as line presentations (1.00° ± 0.58; paired t-test, P = 0.0084), reflecting a perceived increase in the difficulty of aligning the dots reported by many of the subjects (Figure 2a).

**Table 2:** Line and dots - mean errors for vertical and horizontal tasks

		Frame^-18^		Frame°		Frame^+18^	
		line	dot	line	dot	line	dot
		Mean ± SD	Mean ± SD	Mean ± SD	Mean ± SD	Mean ± SD	Mean ± SD
Vertical (n = 30)	S	-0.99 ± 0.89	-1.21 ± 1.75	0.05 ± 0.44	-0.71 ± 0.71	1.06 ± 1.19	0.81 ± 1.00
	US	1.26 ± 0.75	1.78 ± 1.25	0.44 ± 0.31	0.81 ± 0.51	1.34 ± 1.03	1.14 ± 0.80
Horizontal (n = 30)	S	-0.99 ± 1.49	-1.79 ± 1.76	-0.06 ± 0.33	-0.07 ± 0.54	0.55 ± 1.00	1.60 ± 1.64
	US	1.12 ± 1.43	1.95 ± 1.58	0.35 ± 0.26	0.59 ± 0.29	0.82 ± 0.83	1.94 ± 1.24

Comparison of mean signed (S) and unsigned (US) errors (degrees from gravitational vertical and horizontal) for rod as line versus dots (same individual) and SVH versus SVV (different individuals). Values are mean ± standard deviation.

**Figure 3:**
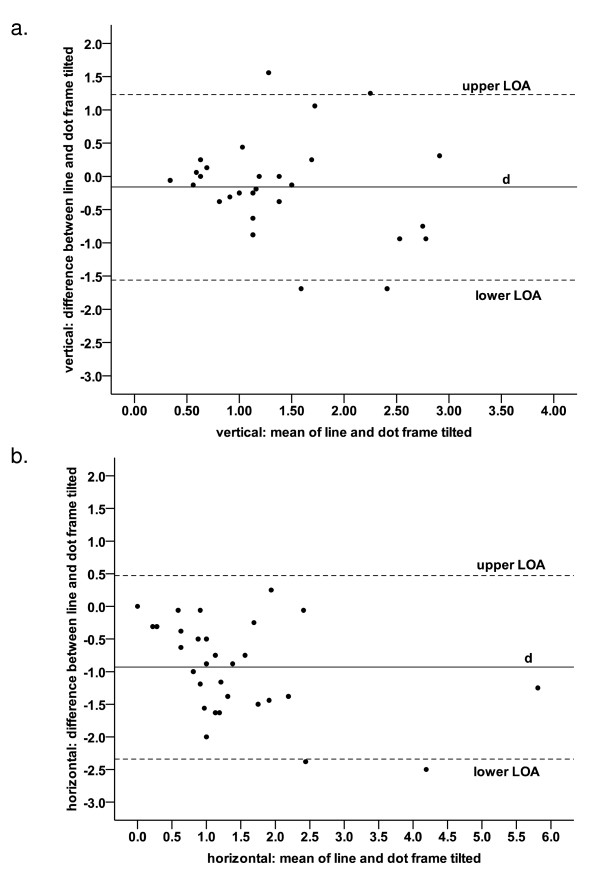
**Bland and Altman plots showing agreement between line and dots**. Bland Altman plots of method agreement comparing the use of a line versus dots to test for subjective visual vertical (a) and horizontal (b) when the frame is tilted (n = 30). Solid line represents the mean difference of errors (d) while the dashed lines represent the upper and lower limits of agreement.

Bland Altman analysis of the combined line and dot data for the frame tilted conditions revealed that the mean difference (d) between the errors for line and dots was -0.16° (CI: -0.43° to 0.10°; Figure 3a). The upper and lower limits of agreement were 1.23° and -1.56° respectively.

### Rod as Line versus Dots - Horizontal

In the test comparing SVH using line versus dots, 16 of the 30 participants were male, mean age 37.74 ± 11.96 years (range: 20-60). All of the errors followed a normal distribution. The signed and unsigned results (mean ± SD) of the trials when the rod was a line compared to two dots are shown in Table 2. The distribution of absolute errors with rod as line presentations had a mode at values < 0.5° (Figure 2b), and did not differ from that found in the previous study (Figure 1c), (rod as line, frame tilted: Z = 1.186, *P *= 0.12).

When SVH was tested using the rod as dots presentation the mode of the absolute error shifted to the 1° bin, and the distribution of the combined (frame^+18 ^and frame^-18^) unsigned errors was similar to that of the rod as dots absolute error for SVV (Figure 2a &2b).

Figure 3b shows the Bland Altman plot of the SVH data, which revealed a fixed error between the line and dot presentations, with a mean difference of -0.93° (CI: -1.20° to -0.67°). The limits of agreement were correspondingly skewed at 0.47° (upper) and -2.34° (lower).

## Discussion

The current study used the CRAF system to investigate the range of errors for both SVH and SVV under two rod conditions. In the first instance, the rod was a line and the results for SVV were similar to those found in previous studies using the CRAF test [17,18] with the majority of individuals having a mean unsigned error of 2.6° or less when the frame was tilted. For SVH the range of errors was skewed towards 0° (gravitational horizontal) and approximately one third of the participants reported using the appearance of the line as a cue when judging horizontal. None of the participants in the SVV test reported this effect. This difference may have been a consequence of the different numbers of pixels along the horizontal and vertical axes of the screen. The chosen solution to this problem was to change the rod to two dots representing the ends of a line.

The Bland Altman analysis showed a high level of agreement for SVV between the results for the dots and line with the mean difference being very close to 0°. For horizontal, the mean difference was nearly 1° indicating a fixed bias between the two methods. This suggest that the results obtained using a line were not a true measure of horizontal perception but were biased by some individuals' familiarity with computer drawn lines.

A frame° condition was included in these tests as a control. The results using the dots were comparable with control subjects in rod only studies (no frame) with a difference of less than 1° in the normal range (mean ± 2SD) of errors [8,10,14,18]. While the two tests are not necessarily equivalent, Bagust [17] found no significant difference between the errors generated under no frame and untilted frame conditions. Previous studies have set an upper limit for the normal range of unsigned errors under rod only conditions, from 2° [16,20] to 3° [3,15] for both SVV and SVH. All participants in this study fell within this range.

Tilting the frame resulted in an increase in the normal range of errors for both SVV (3.96°) and SVH (4.76°). These errors were smaller than those reported in previous studies using mechanical systems which range from 6° [9,11] to 11° [8,10]. This could be a result of the greater accuracy with which the error is measured using the CRAF compared to the other systems. Although some pixilation is visible in the tilted frame when viewed through the eyeglasses and the edges of the screen are detectable, the influence of these effects is thought to be minimal as the CRAF test has been designed with optimal gap size between rod and frame [21] and visual field coverage [22].

When the results for SVV and SVH were compared for the current study, it was found that there were no significant differences between the dot results in either the frame° or frame^-18 ^conditions, as has been found in earlier studies [20,23]. This suggests that representing the rod with two dots gives a better indication of the normal range of perceptual patterns in SVV and SVH.

## Competing interests

The authors declare that they have no competing interests.

## Authors' contributions

JB wrote the Rod and Frame software for this project. Both authors were involved in the design of the study, the data collection, analysis of the data and the drafting of the manuscript. Both authors read and approved the final manuscript.
